# Effect of silymarin and metformin on the sperm parameters and histopathological changes of testes in diabetic rats: An experimental study

**DOI:** 10.18502/ijrm.v19i12.10060

**Published:** 2022-01-12

**Authors:** Bagher Pourheydar, Fatemeh Azarm, Gholamhossein Farjah, Mojtaba Karimipour, Maryam Pourheydar

**Affiliations:** ^1^Department of Anatomical Sciences, Faculty of Medicine, Urmia University of Medical Sciences, Urmia, Iran.; ^2^Faculty of Medicine, Urmia University of Medical Sciences, Urmia, Iran.

**Keywords:** Diabetes, DNA damage, Metformin, Silymarin, Sperm, Testis.

## Abstract

**Background:**

Oxidative stress is a major contributor to diabetes, which can lead to testicular damage and infertility.

**Objective:**

This study aimed to compare the effects of metformin as a chemical drug with silymarin as an herbal agent on the sperm parameters and histopathological changes of testes in diabetic rats.

**Materials and Methods:**

Thirty-two male Wistar rats (250-270 gr) were randomly divided into four groups: 1) control; 2) diabetic; 3) diabetic+metformin 200 mg/kg; and 4) diabetic+silymarin 100 mg/kg. Daily injections were administered intraperitoneally for 56 days. At the end of the treatment, blood sampling was performed for biochemical assessment. Then, the rats were sacrificed and their left testis and epididymis were cut for sperm analysis as well as histopathology and morphometric evaluation.

**Results:**

Diabetes was associated with a reduced sperm count, motility, viability, maturity, and chromatin quality of sperm (p ≤ 0.001). It was also associated with a higher malondialdehide level and lower total antioxidant capacity level of serum in comparison with the control group (p ≤ 0.001). There was a significant difference in the seminiferous tubule diameter, germinal epithelium height, and testicular histopathological alterations in the diabetic rats compared with the control rats (p ≤ 0.001). Treatment with metformin and silymarin improved the above-mentioned parameters and this improvement was more substantial in silymarin-treated animals (p ≤ 0.001).

**Conclusion:**

In diabetic rats, metformin and silymarin improved sperm parameters, sperm DNA integrity, seminiferous tubule diameter, germinal epithelium thickness, and testicular histopathological complications; this improvement was more substantial in the silymarin-treated group. So, the findings of this study suggest that silymarin is more effective than metformin in treating diabetic-induced infertility.

## 1. Introduction

Diabetes mellitus is a metabolic disorder that has several functional and structural complications. Diabetes exerts negative effects on various organs, especially on the testis (1). Diabetic patients can suffer from various genital dysfunctions such as spermatogenesis disorder. A previous study showed that diabetes reduced the sperm parameters and increased the number of abnormal sperms (2). It has been suggested that oxidative stress may play a role in the pathogenesis of diabetes. Thus, in people with diabetes, hyperglycemia causes excessive production of reactive oxygen species (ROS) which leads to modification in proteins and nucleic acids and results in DNA and RNA damage. It also causes testicular apoptosis and decreases the levels of antioxidant enzymes (3). It is believed that antioxidants have a crucial role in reducing the damage of oxidative stress. One of the chemical agents which is widely used for treating type-2 diabetes is metformin (4). It decreases gluconeogenesis in the liver (5), inhibits insulin resistance in the liver and skeletal muscle, and suppresses sugar uptake in the intestines (6). It also possesses anti-inflammatory (7) and antioxidant (8) properties. Metformin, despite having beneficial properties, has several side effects such as gastrointestinal symptoms, the most common of which are diarrhea, heartburn, nausea, abdominal pain, bloating and retching (9). Herbal plants with antioxidant properties and fewer side effects are alternative therapies.

Silymarin, a polyphenolic flavonoid, is extracted from the milk thistle (*Silybum marianum*). Silybin is the main component of silymarin and most of silymarin's phytochemical properties are because of silybin. Several studies have demonstrated the antioxidant, anti-inflammatory, antineoplastic, antifibrotic, and immunomodulatory properties for silymarin (10). Silymarin as an antioxidant reacts against ROS and scavenges free radicals, and improves the antioxidant defense system by strengthening endogenous antioxidant enzymes such as glutathione and superoxide dismutase. The antioxidant and anti-diabetic effects of metformin and silymarin have been previously studied (11). Metformin as a chemical drug, in addition to its beneficial effects, possesses several side effects which negatively affect the patient's quality of life. So, the present study aimed to compare the effects of metformin as a chemical drug and silymarin as an herbal agent on the testis and sperm parameters of diabetic rats.

If the results of this study confirm the positive effects of silymarin on the male reproductive system, it is hoped that, after clinical trials, this agent could be used to improve diabetic-induced complications on sperm parameters and testicular tissue and may be used as an effective agent for infertility treatment.

## 2. Materials and Methods

### Animals

This experimental study was performed on 32 male Wistar rats, aged four months (250-270 gr). The animals were purchased from the animal house of the Faculty of Medicine at Urmia University of Medical Sciences, Urmia, Iran. Animals were housed in plastic cages at 12 hr light/dark cycle and room temperature of 21-23°C and fed with standard commercial laboratory chow and water.

### Experimental design

The animals were randomly divided into four groups (n = 8 in each group) and the drugs were administered daily for 56 days, as follows:

1) Control (intact) group (C) which received intraperitoneally equal volume of saline;

2) Diabetic group (D) which received streptozotocin (STZ) (Sigma, St. Louis, Mo, USA) (60 mg/kg/ip) (12);

3) (D+Met) diabetic group which received STZ+metformin (Merck, Germany) (200 mg/kg/ip) (13); and

4) (D+Sil) diabetic group which received STZ+silymarin (100 mg/kg/ip) (14).

### Induction of diabetes mellitus

Diabetes was induced by a single injection of STZ (60 mg/kg/IP), which was freshly dissolved in cold normal saline. Three days after the STZ injection, hyperglycemia was confirmed by analyzing the tail blood glucose levels with a digital glucometer (Elegance, Model: no: CT-X10 Germany). Rats with high blood glucose levels ( > 250 mg/dl) were considered diabetic.

### Epididymal sperm preparation

Sperms were obtained from the caudal part of the epididymis. For this purpose, rats were sacrificed using ketamine (160 mg/kg, Daroopakhsh, Iran) and xylazine (20 mg/kg, Daroopakhsh, Iran). The abdominal skin was then sterilized with 70% ethanol, an incision was created on the abdomen, and the testes with epididymis were removed. The cauda epididymis of the left testis was removed and minced in 2 ml of human tubal fluid which was previously warmed to 37°C. Then the cauda epididymis was cut into little sections for releasing more sperm. Finally, the sperm suspension was incubated in 37°C and 5% CO ${}_{2}$ for 30 min (15).

### Sperm parameters

The epididymal sperm counting was performed by a hemocytometer (Neubauer chamber) (16). 1 ml of sperm suspension was diluted with human tubal fluid in a ratio of 1 to 20. Then one drop (10 µl) of diluted suspension was transferred into the Neubauer chamber and after five min was counted under a light microscope (Olympus, BH2, Japan). Counting was performed according to the World Health Organization manual. The count of the sperm in the 1 ml sperm suspension was calculated by the following formula:

Sperm cell number = [n.50, 000.d] where n = the counted sperm number, d = reverse of sperm suspension dilution.

Sperm viability was evaluated by eosin-nigrosin staining. 20 µl of sperm suspension was placed on a microscopic slide and stained with 20 µl eosin (Sigma-Aldrich, Germany); after 30 sec 20 µl of nigrosin solution (Sigma-Aldrich, Germany) was added. After preparing the smears and drying the slides, they were observed under a light microscope (x400 magnification). Live sperms remained unstained following staining, whereas those that showed pink coloration were classified as dead. At least 200 sperms were counted from each sample in ten fields randomly, and the percentage of live sperms was recorded (16).

Eosin-nigrosin staining was used to evaluate sperm morphology. Thin smears were prepared, allowed to dry, fixed and stained with eosin and nigrosine. The slides were investigated under a light microscope with a x40 magnification. The presence of one or more abnormality features such as head, tail, and middle piece defects were analyzed. At least 200 sperms were evaluated for morphological abnormalities. The data were presented as a percentage of morphologically normal sperms (16).

For assessment of sperm motility, 10 µl of sperm suspension was placed on a semen analysis chamber. A minimum of ten microscopic fields were assessed to evaluate sperm motility using a light microscope (x400 magnification). The percentage of sperm motility was calculated for the following patterns: progressively motile sperm (PMS) and nonmotile sperm (NMS).

### Sperm DNA integrity 

Sperm DNA integrity was evaluated by acridine orange (AO) staining. Sperm smears were placed on slides, and were air-dried for one hr and then fixed in methanol/acetic acid (Carnoy's fixative) for two hr. Each sample was stained for 10 min in freshly prepared acridine orange (AO; 0.19 mg/ml) in phosphate-citrate buffer (PH4) for five min.

The slides were investigated with an immunofluorescent microscope (Zeiss Company, Germany) with a 460 nm filter. The green-headed sperms were considered to have double-stranded DNA or healthy DNA and sperms with a yellow or red head were marked as having single-stranded DNA or denatured DNA. At least 100 sperms were assessed on each slide and finally, the percentages of healthy and damaged DNA were determined and compared between the groups (17).

### Sperm nuclear maturity 

Aniline blue (AB) staining was used for the assessment of sperm nuclear maturity. At the spermatogenesis stage, histones are replaced by protamine in the chromatin of sperms. This replacement is very important for the stability and density of sperms (16). A large number of lysine amino acids are found in the histone protein structure, and these react with acidic dyes such as AB. So, in AB staining, the sperms with immature nuclei are seen as dark blue due to the presence of histones, while the healthy sperms with mature nuclei are seen as pale.

First sperm smears were prepared and fixed in 3% buffered glutaraldehyde in 0.2 M phosphate buffer for 30 min. Next the smears were stained with 5% aqueous aniline blue in 3% acetic acid for five min. Finally, the slides were washed and surveyed with a light microscope at x100 magnification. The nuclear maturity of 200 sperms per slide was assessed by a light microscope and the percentage of mature sperms was calculated in each animal (18).

### Body weight and testis weight 

Body weight was recorded one day before STZ-injection and at the end of treatment. At the end of the study, rats were anesthetized with ketamine and xylazine, and blood samples were collected from the heart for malondialdehyde (MDA) and total antioxidant capacity (TAC) levels measurement. The right testis was removed and its weight was determined. The testis weight index was calculated in the experimental groups.

### Measurement of serum MDA

MDA is important in the process of lipid peroxidation and is used as an index of oxidative stress. So, to determine the amount of lipid peroxidation, MDA levels were measured in serum samples. To this end, blood samples were taken from the heart. After centrifuging (3000 rpm for 15 min), the blood serum was separated and frozen at -70°C.

The MDA level was evaluated by placing 0.20 ml of the serum into a test tube which contained 3 ml of glacial acid. 1% thiobarbituric acid and 2% NaOH were added to the tube, which was placed in boiling water for 15 min. Then after cooling, a spectrophotometer read the absorbance of the pink-colored product at 532 nm. The calibration curve was constructed by MDA tetrabutylammonium salt obtained from Sigma Company (USA) (19).

### Measurement of serum TAC

TAC was used for evaluating the protective effects of silymarin and metformin on diabetic-induced oxidative stress. TAC was measured in the serum in accordance with the guidelines and protocol of the commercial kit LDN (Labor Diagnostika Nord GmbH and Co KG, Germany).

This method is based on the reduction ability of Fe ${}^{3+}$ . In this procedure, a large amount of Fe ${}^{3+}$ in acidic pH was reversed to Fe ${}^{2+}$ and blue dye was formed. The absorption was measured at a wavelength of 593 nm and read by a spectrophotometer. The amount of TAC was expressed in nmol/ml (20).

### Histopathological assay 

After excision of the left testis, it was placed in 10% formalin for 48 hr to fix its tissue. After tissue processing, 5 µm thick sections were prepared with a rotary microtome (Leica Model RM 2145, Germany). Then the slides were stained with hematoxylin and eosin (H&E) (Merck, Germany) and observed under a light microscope equipped with a Sony camera (Zeiss, Cyber-Shot, Japan) (21). The number of spermatogonia, primary spermatocytes, spermatid, and Leydig cells was examined in the histopathological analysis.

### Morphometric study

The height of germinal epithelium and diameter of 50 seminiferous tubules were randomly examined in each group by using a Motic camera and software. The diameter and germinal epithelial height of seminiferous tubules were measured from the spermatogenic cells on the inner surface of the basement membrane through the most advanced cell types lining the lumen of the tubules (22).

### Ethical considerations

All animal experimentation protocols were carried out under the supervision of the Ethics Committee of Urmia University of Medical Sciences (Code: IR.UMSU.REC.1398.114).

### Statistical analysis

The statistical analysis was performed using the Statistical Package for the Social Sciences software version 16.0 for windows (SPSS Inc, Chicago, II, United States). All data were presented as means ± standard deviation (SD). Analysis of variance (ANOVA) was applied for multiple groups comparison, followed by Tukey's post hoc test. P < 0.05 was considered statistically significant.

## 3. Results

### Sperm count 

The sperm count findings are summarized in table I. According to the data, there was a significantly lower number of sperms in the diabetic rats compared to the control group. Significantly higher numbers of sperm were observed in the D+Met and D+Sil groups in comparison with the diabetic group (p ≤ 0.001, table I). There was also a significant difference in terms of sperm count between the D+Met and D+Sil groups (p ≤ 0.001).

### Sperm motility 

The sperm motility data are also summarized in table I. The sperm motility of rats in the diabetic group decreased significantly in comparison to those of the control group (p ≤ 0.001). In treated groups (groups that received metformin and silymarin), the sperm motility improved significantly in comparison to those of the diabetic group (p ≤ 0.001). A significant difference was observed between the D+Met and D+Sil groups (p ≤ 0.001).

### Sperm morphology

According to the data of table I, the rats in the diabetic group had significantly fewer sperms with normal morphology than the control group (p ≤ 0.001). Statistical analysis showed that there was a significant difference in the percentage of sperms with normal morphology between the diabetic rats and those in the D+Met and D+Sil groups (p ≤ 0.001).

The morphological observation indicated that treatment with silymarin significantly (p ≤ 0.001) reversed the sperm morphological anomalies and significantly increased the percentage of normal sperms compared to the untreated group so that there was no significant difference between the control and D+Sil groups (p = 1.00).

### Sperm viability

The percentage of live sperms in the diabetic group was significantly lower (p ≤ 0.001) in comparison with the control group. Diabetic rats which were treated with metformin or silymarin showed a considerably higher percentage of live sperms when compared to the diabetic group (p < 0.05) (Table I).

### Sperm DNA integrity

The analysis of sperm DNA integrity is presented in table I. The findings indicated that diabetes increased the percentage of red spermatozoa (single-stranded DNA sperm) compared to the control group. Treatment with metformin and silymarin significantly decreased the percentage of spermatozoa with DNA damage in comparison with the diabetic group (p ≤ 0.001). There was also a significant difference in the percentage of single-stranded DNA sperm in the D+Sil group compared to the D+Met group (p ≤ 0.001).

### Sperm nuclear maturity

AB staining revealed that diabetes was associated with asignificantly higher percentage of dark blue stained sperms (sperms with an immature nucleus) compared to the control group (p ≤ 0.001) (Table I). Statistical analysis indicated that treatment with silymarin remarkably decreased the percentage of sperms with chromatin abnormalities compared to the diabetic group (p ≤ 0.001). Metformin had no significant effect on the histo-protamine replacement in the sperm maturation process in comparison with the diabetic group (p = 0.43).

### MDA measurement in serum

According to the results shown in table I, the concentration of plasma MDA was considerably higher in diabetic rats compared to the control group (p ≤ 0.001). Treatment with metformin and silymarin seemed to significantly reduce the level of serum MDA (p ≤ 0.001) compared to the diabetic group. Also, a significant difference was observed between the D+Met and D+Sil groups in terms of MDA concentration (p ≤ 0.001).

### TAC measurement in serum

The concentration of serum TAC was significantly lower in the diabetic group compared to the control group (p ≤ 0.001) (Table I). Statistical analysis indicated that the serum TAC was remarkably improved in the D+Sil group in comparison with the diabetic group (p ≤ 0.001). Metformin had no significant effect on the serum level of TAC when compared with the diabetic group (p = 1.00). Also, the mean concentration of TAC was significantly higher in the D+Sil group as compared with the D+Met group (p ≤ 0.001).

### Body weight and testis weight

The effects of the various treatments on the rats' body and testis weight and index of testis weight are presented in table II. Statistical analysis revealed that there was no significant difference between the experimental groups in terms of initial body weight (p = 1.00). At the end of the study a remarkably lower body weight was found in the diabetic group compared to the control group (p ≤ 0.001). A partial recovery in body weight was observed in animals treated with metformin and silymarin and there was a significant difference in body weight between the diabetic and treated animals (p ≤ 0.001). In addition, a significant difference was seen between the D+Met and D+Sil groups in terms of final body weight (p ≤ 0.001). Moreover, a considerably lower testis weight was observed in the diabetic group compared to the control group (p ≤ 0.001), while the findings indicated that treatment with metformin and silymarin resulted in a significant rise in testis weight (p ≤ 0.001) when compared with the diabetic group. Further, there was a significant difference between the D+Met and D+Sil groups in terms of testis weight (p ≤ 0.001).

### Effect of metformin and silymarin on seminiferous tubule diameter and germinal epithelium height 

**Figure 1:**
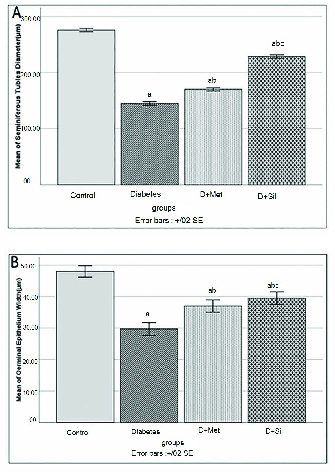
Effect of metformin (Met) and silymarin (Sil) on A) Seminiferous tubule diameter and B) Germinal epithelium height in different groups. The data are expressed as Mean ± SD. a: Significantly different from the control group, b: Significantly different from the diabetic group, c: Significantly different from the D+Met group.

Figure 1 shows the diameter and germinal epithelial height of seminiferous tubules for all of the groups. The mean diameter of seminiferous tubules and germinal epithelial height in the diabetic group decreased significantly in comparison to those of the control group (p ≤ 0.001), while the means of these parameters were significantly improved in animals treated with metformin and silymarin in comparison to those of the diabetic group (p ≤ 0.001).

### Effect of metformin and silymarin on spermatogenic cell count

**Figure 2:**
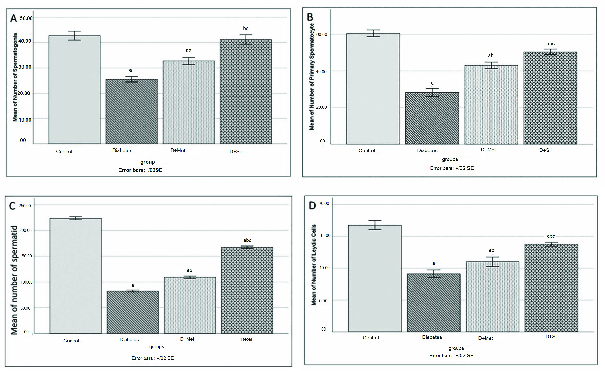
Effect of metformin (Met) and silymarin (Sil) on the number of A) Spermatogonia, B) Primary spermatocytes, C) Spermatids and D) Leydig cells in different groups. The data are expressed as Mean ± SD. a: Significantly different from the control group, b: Significantly different from the diabetic group, c: Significantly different from the D+Met group.

The mean number of spermatogenic cells is shown in figure 2. According to figure 2A, significantly fewer spermatogonia, primary spermatocytes, spermatid, and Leydig cells were seen in the diabetic group compared to the control group (p ≤ 0.001). The findings indicated that treatment with metformin and silymarin significantly increased the above-mentioned cell counts (p ≤ 0.001).

### Histopathological findings

**Figure 3:**
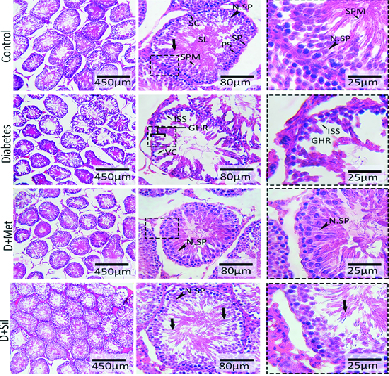
Cross-sections of testicular tissue from different groups. Normal cell distribution, spermatogenesis (N.SP), and spermiogenesis (thick arrow) can be seen in the control group. The section from the diabetes group shows germinal epithelium height reduction (GHR), incomplete spermatogenic series (ISS), vacuolation (VC), germ cell dissociation, and irregular germ epithelium arrangement, which are ameliorated in the D+Met and D+Sil groups. SPM: Spermatid, PS: Primary spermatocyte, SP: Spermatogonia, SC: Sertoli cell, SL: Seminiferous tubule lumen. (Hematoxylin and eosin, x100, x400, and x800 magnification).

The histopathological analyses of the testes indicated that the seminiferous tubules in the control group had normal morphology, the germinal epithelium in these tubules contained all of the spermatogenic line cells such as spermatogonia, primary spermatocytes and spermatids, and the lumen of the seminiferous tubules contained a large number of spermatozoid. The basement membrane had a normal thickness and the interstitial spaces were filled by several Leydig cells (Figure 3). Compared to the control group, in the diabetic group, disorganization and deformation of the seminiferous tubules were observed (Figure 3). In addition, the basement membrane was thicker in the diabetic group. Moreover, in the diabetic group, degeneration of germinal epithelium cells, atrophy of Leydig cells, and reduced spermatozoid number in the tubule lumen were also observed. Treatment with metformin and silymarin seemed to significantly reduce the diabetic histopathologic complications (Figure 3).

**Table 1:** Effects of metformin and silymarin on sperm count, motility, morphology, viability, maturity and DNA quality, and MDA and TAC concentrations in the different groups


**Variable**	**Control**	**Diabetes**	**D+Met**	**D+Sil**	p-value*
**Sperm count (ml × 10^6^)**	116.0 ± 2.78 (0.98)	34.5 ± 2.07 ${}^{a}$ (0.73)	42.0 ± 2.07 ${}^{a}{}^{b}$ (0.73)	54.0 ± 2.00 ${}^{a}{}^{b}{}^{c}$ (0.71)	≤ 0.001
**Motile sperm (%)**	62.00 ± 3.38 (1.20)	22.63 ± 2.77 ${}^{a}$ (0.98)	46.38 ± 2.67 ${}^{a}{}^{b}$ (0.94)	58.13 ± 3.31 ${}^{b}{}^{c}$ (1.17)	≤ 0.001
**Sperm with normal morphology (%)**	86.00 ± 2.56 (0.91)	48.00 ± 2.73 ${}^{a}$ (0.96)	77.75 ± 2.38 ${}^{a}{}^{b}$ (0.84)	84.50 ± 2.14 ${}^{b}{}^{c}$ (0.76)	≤ 0.001
**Viable sperm (%)**	89.63 ± 2.97 (1.05)	42.38 ± 2.77 ${}^{a}$ (0.98)	55.13 ± 2.64 ${}^{a}{}^{b}$ (0.93)	87.50 ± 2.45 ${}^{b}{}^{c}$ (0.87)	≤ 0.001
**Sperm with DNA damage**	1.00 ± 0.76 (0.27)	33.25 ± 2.82 ${}^{a}$ (1.00)	25.50 ± 2.20 ${}^{a}{}^{b}$ (0.78)	7.75 ± 2.19 ${}^{a}{}^{b}{}^{c}$ (0.77)	≤ 0.001
**Sperm with immature nucleus (%)**	7.00 ± 1.77 (0.63)	41.50 ± 2.51 ${}^{a}$ (0.89)	39.00 ± 2.27 ${}^{a}$ (0.81)	33.13 ± 2.17 ${}^{a}{}^{b}{}^{c}$ (0.77)	≤ 0.001
**MDA level in serum (nmol/ml)**	1.2 ± 0.18 (0.06)	4.5 ± 0.15 ${}^{a}$ (0.05)	3.7 ± 0.15 ${}^{a}{}^{b}$ (0.05)	2.1 ± 0.22 ${}^{a}{}^{b}{}^{c}$ (0.08)	≤ 0.001
**TAC level in serum (nmol/ml)**	0.80 ± 0.12 (0.04)	0.28 ± 0.02 ${}^{a}$ (0.01)	0.32 ± 0.03 ${}^{a}$ (0.01)	0.58 ± 0.03 ${}^{a}{}^{b}{}^{c}$ (0.01)	≤ 0.001
Data are presented as Mean ± SD (SE). ${}^{a}$ Significantly different from the control group, ${}^{b}$ Significantly different from the diabetic group, ${}^{c}$ Significantly different from the D+Met group, *One Way-ANOVA test, D: Diabetes, Met: Metformin, Sil: Silymarin, MDA: Malondialdehyde, TAC: Total antioxidant capacity					

**Table 2:** Effects of metformin and silymarin on body and testis weights and index of testis weight in different groups


**Variable**	**Control**	**Diabetes**	**D+Met**	**D+Sil**	p-value*
**Initial body weight (gr)**	255.00 ± 7.03 (2.49)	256.13 ± 6.81 (2.41)	254.88 ± 6.79 (2.40)	256.38 ± 7.96 (2.82)	1.00
**Final body weight (gr)**	266.38 ± 8.11 (2.87)	158.25 ± 8.71 ${}^{a}$ (3.08)	180.00 ± 7.33 ${}^{a}{}^{b}$ (2.59)	218.00 ± 6.16 ${}^{a}{}^{b}{}^{c}$ (2.18)	≤ 0.001
**Testis weight (gr)**	1.69 ± 0.04 (0.01)	0.90 ± 0.03 ${}^{a}$ (0.01)	1.10 ± 0.09 ${}^{a}{}^{b}$ (0.03)	1.36 ± 0.02 ${}^{a,b,c}$ (0.01)	≤ 0.001
**Index of testis weight (%)**	0.0063 (0.63 %)	0.0056 (0.56 %)	0.0061 (0.61 %)	0.0062 (0.62 %)	-
Data are presented as Mean ± SD (SE). ${}^{a}$ Significantly different from the control group, ${}^{b}$ Significantly different from the diabetic group, ${}^{c}$ Significantly different from the D+Met group, *One Way-ANOVA test, D: Diabetes, Met: Metformin, Sil: Silymarin					

## 4. Discussion

The present study aimed to investigate the effects of silymarin in comparison with metformin on testis and sperm parameters in diabetic rats. Our data demonstrated that diabetes significantly reduced the body and testis weight, sperm parameters, sperm DNA integrity, seminiferous tubule diameter, number of spermatogenic cells and TAC level and increased the MDA level in comparison with the control group; the use of metformin and silymarin markedly improved the aforementioned parameters (p ≤ 0.001). These findings were consistent with those of other research.

A previous study (23) investigated the effect of metformin and *Nigella sativa* on the reproductive system in diabetic male rats. Its findings indicated that diabetes reduced the relative weight of testes and the level of testosterone, and co-administration of metformin and *Nigella sativa* oil resulted in the improvement of the mentioned parameters. Other studies have revealed that diabetes-induced oxidative stress decreases the levels of antioxidant enzymes in Leydig cells which could result in the reduction of testosterone synthesis. The low level of testosterone leads to the dysfunction of spermatogenesis, degeneration of seminiferous tubules, and, finally, decreased testis weight. In the present study, it seems that metformin and silymarin caused an increase in serum testosterone levels which resulted in the improvement of count and function of germinal cells and finally, the rise of testis weight.

Another study examined the effect of silymarin and silibin in STZ-induced diabetic rats (24). Its findings showed a significant reduction in the body weight of the diabetic group. Also, in the animals treated with silymarin, body weight was significantly increased when compared with the diabetic group. The data of our study also showed that silymarin normalized the reduced testicular weight in the diabetic group. It seems that silymarin stimulated germ cells' survival and spermatogenesis via the alleviation of oxidative stress and protected the rats from testicular weight loss (25).

Hyperglycemia is one of the major factors which results in infertility. A previous study showed that oxidative stress induced by hyperglycemia can have defective effects on the reproductive system such as sexual organ weight reduction, testicular atrophy, and decreased sperm count (26). The sperm cell membrane contains a large number of polyunsaturated fatty acids, which makes them very sensitive to oxidative damage (27). So, lipid peroxidation affects the integrity of the sperm cell's membrane and causes a decrease in sperm motility as well as viability and, finally, can lead to apoptosis and reduced sperm count (28). Therefore, antioxidant administration could be one of the beneficial methods for preventing diabetes-induced infertility. Silymarin, as a natural polyphenolic compound, can react with ROS and free radicals and change them into compounds with less toxicity. It also strengthens the effects of antioxidant enzymes such as glutathione and superoxide dismutase (27).

The effect of silymarin on methotrexate-induced testicular damage was studied by researchers (25). They reported that methotrexate caused a loss of germ cells and sperms and reduced antioxidant enzymes. It also decreased sperm motility and viability. Silymarin administration significantly improved these parameters. These findings were in agreement with our study data. The present study indicated that diabetes significantly decreased sperm count, motility and viability, and silymarin administration considerably improved these parameters. The protective effect of silymarin can be explained by its protection of the cell membrane against oxidative stress. The antioxidant effect of silymarin may be due to the components present in silymarin such as silybin, silydianin, silychristin, and flavonolignans.

The findings of our study showed that the serum MDA levels were considerably increased and the TAC levels were decreased in the diabetic rats; treatment with metformin and silymarin improved these levels. These results were in line with previous studies. A recent study surveyed the effect of metformin on diabetic nephropathy (5). Its results indicated that diabetes increased MDA levels and decreased superoxide dismutase levels in kidney tissue, and metformin improved these levels. The findings revealed that metformin inhibited oxidative stress in diabetic rats.

Researchers studied the effect of silymarin on oxidative stress in cadmium-treated mice (29). Their data showed that silymarin reversed the toxicity of cadmium, decreased the lipid peroxidation, and increased the antioxidant enzyme activity. Silymarin is a polyphenolic compound with antioxidant effects. It increases the phosphorylation level of tyrosine or serine residues of nuclear factor erythroid-2-related factor 2, promotes the expression of antioxidant enzymes, and improves the capacity of the antioxidant defense system (30).

Regarding the AB and AO staining, the present study demonstrated that diabetes led to an elevated number of sperms with an immature nucleus and an increased number of sperms with abnormal single-stranded DNA; treatment with silymarin reduced sperm DNA and chromatin disorders. A previous study indicated that over-generation of ROS in diabetes led to defects in sperm chromatin and DNA (31). The integrity of sperm chromatin is an important factor in the fertilization process. Several studies have revealed that increased histone remnants and protamine deficiency in sperm leads to a defect in chromatin condensation, which results in fertilization failure (32). In our study, it seems that silymarin with its antioxidant properties could protect the sperm against diabetic-induced ROS and restore sperm nuclear maturity and sperm DNA integrity.

The present study showed that diabetes resulted in the degeneration of seminiferous tubules, decreased germinal epithelium height, and a reduced number of spermatogonia, spermatocytes, spermatids, and Leydig cells, while treatment with metformin and silymarin improved these histological alterations. It seems that the excessive generation of ROS in diabetic rats destroyed the synthesis of DNA and RNA in the sperm as well as in sexual germ cells, interrupted their divisions and differentiation, and finally, led to a reduction in the number of spermatogenic cells (33).

A previous study investigated the effect of L-carnitine on the histopathology of testes in diabetic rats (22). Its findings showed that diabetes caused deformity of sperms and reduction in the number of spermatogenic cells, and L-carnitine improved these alterations. Since sperms lose a large volume of their cytoplasm during spermatogenesis, they have a low amount of cytoplasmic enzymes, making the sperm's membrane sensitive to ROS (34). An increase in ROS and lipid peroxidation results in seminiferous tubule atrophy and apoptosis of germ cells. In the present study, the improvement of histological alteration can be attributed to the antioxidant properties of metformin and silymarin.

A recent study revealed that diabetes can induce apoptosis in spermatogenic line cells (35). One of the factors which can induce apoptosis is defect in the synthesis of testosterone by Leydig cells. Diabetes-induced ROS affects the Leydig cells' function and reduces the level of testosterone. The integrity of germinal cells and their normal function for mitotic divisions depends on testosterone secretion (36).

Another study examined the effect of metformin and honey on the testes of diabetic rats; the results indicated that diabetes reduced testosterone, luteinizing hormone, and follicle-stimulating hormone levels in the serum and decreased the seminiferous tubule diameters; treatment with metformin and honey improved the levels of the sexual hormones and the histomorphometric alteration in the testes (2). Consistent with other studies, it seems that metformin and silymarin prevented the accumulation of free radicals, restored the integrity of sperm cell membranes, prevented the apoptosis of germ cells, and increased the number of spermatogenic line cells and diameter of seminiferous tubules in the testes of the diabetic rats.

The data of the present study showed that both metformin and silymarin could improve the sperm parameters and testicular histopathological alterations in diabetic rats, but silymarin was more effective. Further research is required for describing the mechanism of their protective efficacy. Similar to our study, researchers investigated the effect of metformin and curcumin on oxidative stress and diabetic nephropathy in rats (37). They reported that curcumin was found to be more effective than metformin in attenuating oxidative stress in diabetic nephropathy.

Finally, the findings of this study suggested that metformin and silymarin have beneficial effects in preventing and treating the complications of diabetes and infertility, and silymarin was found to be more effective.

## 5. Conclusion

The data of this study indicated that metformin and silymarin significantly improved the sperm parameters, sperm DNA integrity and histopathological alterations in diabetic rats, and this improvement was more substantial in silymarin-treated animals. Therefore, it seems that silymarin plays a more effective role than metformin in diabetes-induced infertility. However, further detailed studies are required to investigate the effects of these compounds in more depth.

##  Conflict of Interest

The authors declare no conflict of interest in the present study.
